# Deciphering the complex circulating immune cell microenvironment in chronic lymphocytic leukaemia using patient similarity networks

**DOI:** 10.1038/s41598-020-79121-4

**Published:** 2021-01-11

**Authors:** Zuzana Mikulkova, Gayane Manukyan, Peter Turcsanyi, Milos Kudelka, Renata Urbanova, Jakub Savara, Eliska Ochodkova, Yvona Brychtova, Jan Molinsky, Martin Simkovic, David Starostka, Jan Novak, Ondrej Janca, Martin Dihel, Pavlina Ryznerova, Lekaa Mohammad, Tomas Papajik, Eva Kriegova

**Affiliations:** 1 Department of Immunology, Faculty of Medicine and Dentistry, Palacký University and University Hospital Olomouc, Hnevotinska 3, 775 15 Olomouc, Czech Republic; 2 Department of Hematology-Oncology, Faculty of Medicine and Dentistry, Palacký University and University Hospital Olomouc, Olomouc, Czech Republic; 3 Department of Computer Science, Faculty of Electrical Engineering and Computer Science, VSB-Technical University of Ostrava, Ostrava, Czech Republic; 4 Department of Internal Medicine, Hematology and Oncology, University Hospital Brno and Faculty of Medicine Masaryk University, Brno, Czech Republic; 5 1st Department of Medicine - Department of Hematology, First Faculty of Medicine, Charles University and General University Hospital in Prague, Praha, Czech Republic; 6 Department of Internal Medicine - Hematology, University Hospital and Medical School Hradec Kralove, Hradec Kralove, Czech Republic; 7 Department of Clinical Hematology, Hospital in Havirov, Havirov, Czech Republic; 8 Department of Internal Medicine - Hematology, Third Faculty of Medicine, Charles University and Faculty Hospital Kralovske Vinohrady, Praha, Czech Republic; 9 Department of Hematology and Oncology, University Hospital Plzen, Plzen, Czech Republic; 10 Laboratory of Molecular and Cellular Immunology, Institute of Molecular Biology NAS RA, Yerevan, Armenia

**Keywords:** Cancer, Computational biology and bioinformatics, Immunology, Medical research

## Abstract

The tissue microenvironment in chronic lymphocytic leukaemia (CLL) plays a key role in the pathogenesis of CLL, but the complex blood microenvironment in CLL has not yet been fully characterised. Therefore, immunophenotyping of circulating immune cells in 244 CLL patients and 52 healthy controls was performed using flow cytometry and analysed by multivariate Patient Similarity Networks (PSNs). Our study revealed high inter-individual heterogeneity in the distribution and activation of bystander immune cells in CLL, depending on the bulk of the CLL cells. High CLL counts were associated with low activation on circulating monocytes and T cells and vice versa. The highest activation of immune cells, particularly of intermediate and non-classical monocytes, was evident in patients treated with novel agents. PSNs revealed a low activation of immune cells in CLL progression, irrespective of *IgHV* status, Binet stage and *TP53* disruption. Patients with high intermediate monocytes (> 5.4%) with low activation were 2.5 times more likely (95% confidence interval 1.421–4.403, *P* = 0.002) to had shorter time-to-treatment than those with low monocyte counts. Our study demonstrated the association between the activation of circulating immune cells and the bulk of CLL cells. The highest activation of bystander immune cells was detected in patients with slow disease course and in those treated with novel agents. The subset of intermediate monocytes showed predictive value for time-to-treatment in CLL.

## Introduction

Chronic lymphocytic leukaemia (CLL) is a lymphoproliferative disorder characterised by the progressive accumulation of mature CD19 + CD5 + B cells in the peripheral blood, bone marrow and secondary lymphoid organs^1^. CLL is characterised by an extremely variable clinical course, ranging from a slow and stable disease, which has no requirement for therapy, to an extremely aggressive disease that requires treatment.

While certain intrinsic factors, mainly genomic alterations of CLL cells, have been proven to be crucially involved in CLL pathogenesis, the effect of extrinsic factors, such as direct interactions of CLL cells within the microenvironment, have not been fully estimated. In fact, CLL is highly dependent on bidirectional cross-talk between CLL cells and a supportive microenvironment, which allows CLL cells to evade immunosurveillance^2,3^. Many cellular and molecular players in the tissue microenvironment promote survival, proliferation and resistance of CLL cells, including monocyte-derived nurse-like cells, macrophages and mesenchymal stromal cells^2,4^. Furthermore, the critical importance of the tissue microenvironment is supported by the clinical effects of novel agents that disrupt the interaction between neoplastic cells and the tissue microenvironment^5–7^. Unlike the tissue microenvironment, the complex architecture of the circulating blood microenvironment in the CLL has yet to be described. Studies focused on individual cell populations in blood reported disturbances in the activation and exhaustion of T cells^8,9^, neutrophils^10^, decreased degranulation and increased sensitivity to activation-induced NK cell death^11^, as well as altered composition and phagocytosis of monocytes in patients with CLL^12,13^. Comprehensive studies on circulating immune cells and their cross-talk with neoplastic CLL cells may contribute to new insights into the complexity of tumour immunology in CLL.

Therefore, we aimed to comprehensively characterise circulating immune cells that shape the CLL blood microenvironment, together with CLL cells, in a large real-world cohort of CLL patients, from seven centres in the Czech Republic. Moreover, we addressed the question of whether blood immune cells and their activation differ between patients with varying tumour burdens, clinical course and treatment, with a particular emphasis on the use of novel drugs (ibrutinib, idelalisib and venetoclax).

## Materials and methods

### Patients

Peripheral blood samples were collected from 244 patients with CLL (105 females and 139 males, with a median age of 67 years), from seven haematology-oncology centres in the Czech Republic. The diagnosis of CLL was established according to the International Workshop on Chronic Lymphocytic Leukemia guidelines^14^. All incoming CLL samples from all centres were included because they all exceeded the limit for minimal residual disease (1 CLL cell per 10,000 leukocytes)^15^. Of the enrolled patients, 123 were treatment-naïve, 67 patients were treated with novel agents (ibrutinib, idelalisib and venetoclax), and 54 patients were previously receiving immunochemotherapy. Detailed clinical characteristics of the enrolled patients are shown in Table 1. Time-to-treatment (TTT) was available for all patients for at least one year of post-sampling follow-up. The study included 52 age-matched healthy control subjects (28 females and 24 males, with a median age of 68 years) selected from members of medical staff or their relatives, where cancer, autoimmunity and chronic diseases were excluded by the questionnaires.

**Table 1:** Characteristics of CLL patients at sampling.

Parameters	CLL patients (n = 244)
Age in years: median (range)	67 (38–89)
Gender: female/male	105/139
Binet stage: A/B/C/n.a.	143/54/44/3
Bulky lymphadenopathy ≥ 5 cm: yes/no/n.a.	75/158/11
Splenomegaly: yes/no/n.a.	88/148/8
*IgHV* mutational status: unmutated/mutated/n.a.	150/83/11
β2-microglobulin (≥ 3.5 mg/dL): yes/no/n.a.	116/109/19
**Genetic characteristics**	
*TP53* disruption (deletion 17p and/or *TP53* mutations): yes/no/n.a.	59/183/2
del(11q22): yes/no/n.a.	55/189/0
del(13q14): yes/no/n.a.	112/81/51
Treatment history: yes/no	121/123
**Patients previously treated with immunochemotherapy**	54
Number of previous treatment regimens: median (range)	1 (1–4)
Time from the last treatment to sampling: median (range) in months	24 (1–173)
**Patients treated with novel drugs**	67
Ibrutinib/idelalisib/venetoclax^#^	42/19/6
Number of previous treatment regimens: median (range)	2 (0–10)
Treatment duration on novel drugs: median (range) in months	9 (1–42)
**CLL cells in peripheral blood**	
Percentage: median (range)	59.5 (0.01–95.9)
CLL cell counts (× 10^9^/L): median (range)	15.8 (0.01–581)

n.a., not available; ^#^5 patients treated with venetoclax were ibrutinib-resistant; Next-generation sequencing was used to detect *TP53* mutations, Sanger sequencing for *IgHV* mutational status and cytogenetics and FISH analysis for deletion 17p and other abberations, as previously reported^16–18^.

All CLL patients and healthy controls provided written informed consent for the use of peripheral blood for research purposes, in accordance with the Declaration of Helsinki. The local ethical committee of the University Hospital and Palacký University Olomouc approved the study. All experiments were performed in accordance with relevant guidelines and regulations.

### Flow cytometry

Six-colour flow cytometry was used to analyse the circulating immune cells and their main activation markers (see the online supplement, Table S1) in the whole blood, as reported previously^10^. All blood samples were processed within 24 h based on the current recommendation for sample processing in multicentric studies^19^ and stability experiments were performed for investigated populations and activation markers. Whole blood was incubated with the mix of conjugated antibodies for 20 min in the dark, at room temperature. Then, the red blood cells were lysed with 2 mL of FACS lysing solution (diluted 1:10 with distilled water; Becton–Dickinson, San Jose, CA, USA) and washed with phosphate-buffered saline. The main blood cell populations were identified using a sequential gating strategy^20^, after the exclusion of doublets (FSC-A versus FSC-H), as follows: CLL cells (CD5+/CD19+), CD4+ T cells (CD3+/CD4+), CD8+ T cells (CD3+/CD8+), Treg cells (CD4+/CD25+/CD127−), NK cells (CD3−/CD16+/CD56+), neutrophils (CD15+/CD16+) and monocytes (CD14+). Monocyte subsets were gated as follows: classical (CD14+/CD16−), intermediate (CD14+/CD16+) and non-classical (CD14dim/CD16+). The complete list of used antibodies (all BioLegend, San Diego, CA, USA) and their combinations is displayed in the online supplement (Table S2). Isotype-matched FITC, PE, PerCP-Cy5.5, PE-Cy7, APC and APC-Cy7-conjugated irrelevant antibodies (all MOPC-21, BioLegend) were used as negative controls. The analysis was performed using a flow cytometer BD FACSCanto II (Becton Dickinson). Flow cytometry data was analysed using the FlowJo vX0.7 software (Tree Star, Inc., San Carlos, CA, USA).

The main populations (lymphocytes, neutrophils, monocytes) were calculated as a percentage of immune cell singlets. The percentages of subpopulations were calculated as part of the parental populations: CLL from lymphocytes, CD4+ and CD8+ T cells from CD3+ T cells, Treg cells from CD4+ T cells and classical/intermediate/non-classical subsets from monocytes, respectively. In all experiments, a minimum of 20,000 events was counted for lymphocyte population, a cut-off of 500 events was used to evaluate activation markers in monocyte subsets and NK cells.

CLL and immune cell counts were expressed as the number of cells per unit of volume (cells/L) and were calculated by multiplying a percentage of cells derived from the flow cytometry by the number of white blood cells from a complete blood count measured within two hours after sampling by an automated haematological analyser. Studied subpopulations and markers were expressed as a percentage of positive cells and median fluorescence intensity (MFI).

### Statistics and patient similarity network analysis

Statistical tests, including the non-parametric Mann–Whitney–Wilcoxon test, Spearman's Rank-Order Correlations, generation of Receiver Operating Curves (ROC), Kaplan–Meier (K–M) curves, and odds ratio (OR), were performed using R statistical software (http://www.r-project.org/). P-values of < 0.05 were considered significant.

For the clustering of patients and visualisation of patient similarities on the basis of circulating cell populations, a method of network (Patient Similarity Network, PSN) construction based on nearest-neighbour analysis was applied^21–23^. The data on the activation of individual cell populations and/or their counts were transformed into the network, followed by the automatic detection of clusters in the network^24^. To convert the dataset to a weighted undirected network, the LRNet method^21^ based on nearest-neighbour analysis was used (https://homel.vsb.cz/~kud007/lrnet_files/). Modularity and silhouettes were used to assess the quality of the obtained networks and clusters. For a detailed description of the methodology used, see the online supplement.

Next, the trends of changes in activation of immune cells across particular clusters were calculated. Using the visualised layout of PSNs, we added one additional marker to each patient (= network vertex) related to its horizontal position in this layout. In our case, it was an x-coordinate (the unit is a pixel), where the x-coordinate equals to zero in the center of the layout. Patients to the left of this centre have a negative x-coordinate and patients to the right have a positive x-coordinate. Then, scatterplots expressing the relationships between investigated activation markers and the patient's horizontal position in the layout were obtained. For more information on PSN construction and trend visualisation, see the online supplement.

### Ethics approval and consent to participate

This study was approved by the ethical committee of the University Hospital and Palacký University Olomouc. All CLL patients and healthy controls provided written, informed consent for the use of peripheral blood for research purposes, in accordance with the Declaration of Helsinki.

## Results

### Analysis of the relationship between the activation status of immune cells and CLL cell counts

First, we investigated the relationship between circulating immune cell populations, their activation and the number of CLL cells using correlation analysis and heat maps.

Correlation analysis revealed a strong negative correlation of CLL cell counts with HLA-DR and CD64 expression on classical and intermediate monocytes, HLA-DR on non-classical monocytes, CD4+ and CD8+ T cells, as well as CD69 on NK cells in CLL patients (*P* < 0.001) (online supplement, Table S3). Additionally, CLL counts correlated positively with Tregs and CD4+ /CD8+ ratio (both *P* < 0.001). The number of CLL cells did not correlate with the number of classical monocytes (*P* = 0.480), intermediate monocytes *(P* = 0.880) or non-classical monocytes (*P* = 0.120), respectively (see the online supplement Table S3).

**Figure 1:**
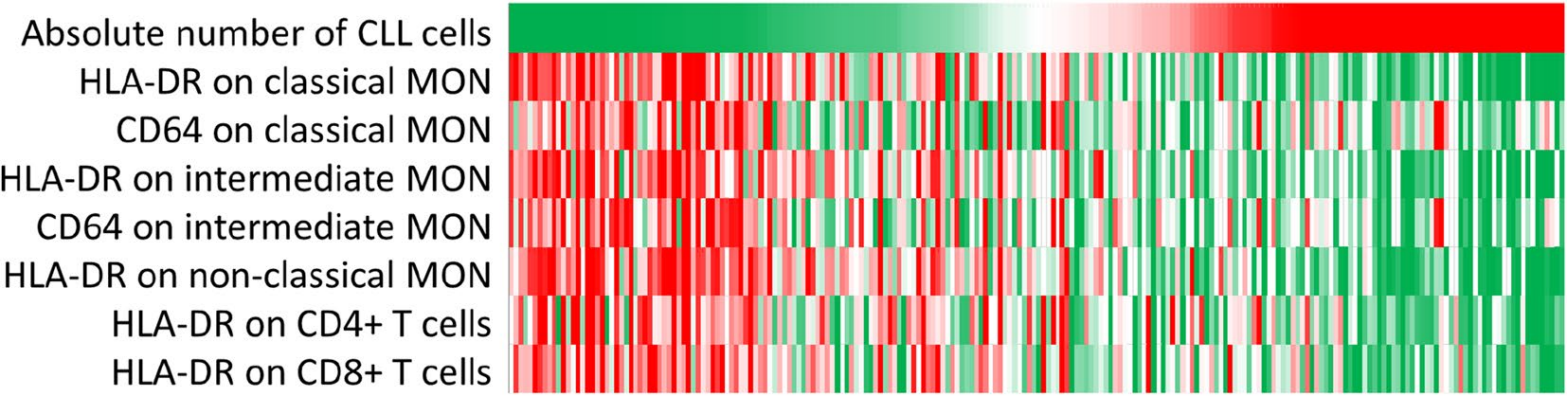
Heat map illustrating the relationship between expression of activation markers on immune cells and the CLL cell counts. Each column represents an individual CLL patient; the rows represent the CLL cell counts (× 10^9^cells/L) or the expression levels of studied markers, respectively. Each cell is coloured based on the CLL cell counts or the level of expression (MFI) of HLA-DR and CD64 on the populations in that patient, from the lowest (green) to the highest (red) values.

Further, heat map analysis was used to visualise the relationships between CLL cell counts and activation of cell subsets. As shown in Fig. 1, the patients with high CLL cell counts had less activated immune cell microenvironment than those with lower CLL cell counts.

### Patient similarity network analysis visualised the relationship between the activation status of immune cells and CLL cell counts

To achieve a better understanding and more in-depth analysis of the associations between immune cell microenvironment and bulk of CLL cells revealed from heat maps, which are insufficient in the visual exploration of multiple associations, we analysed the data using multivariate PSNs.

**Figure 2:**
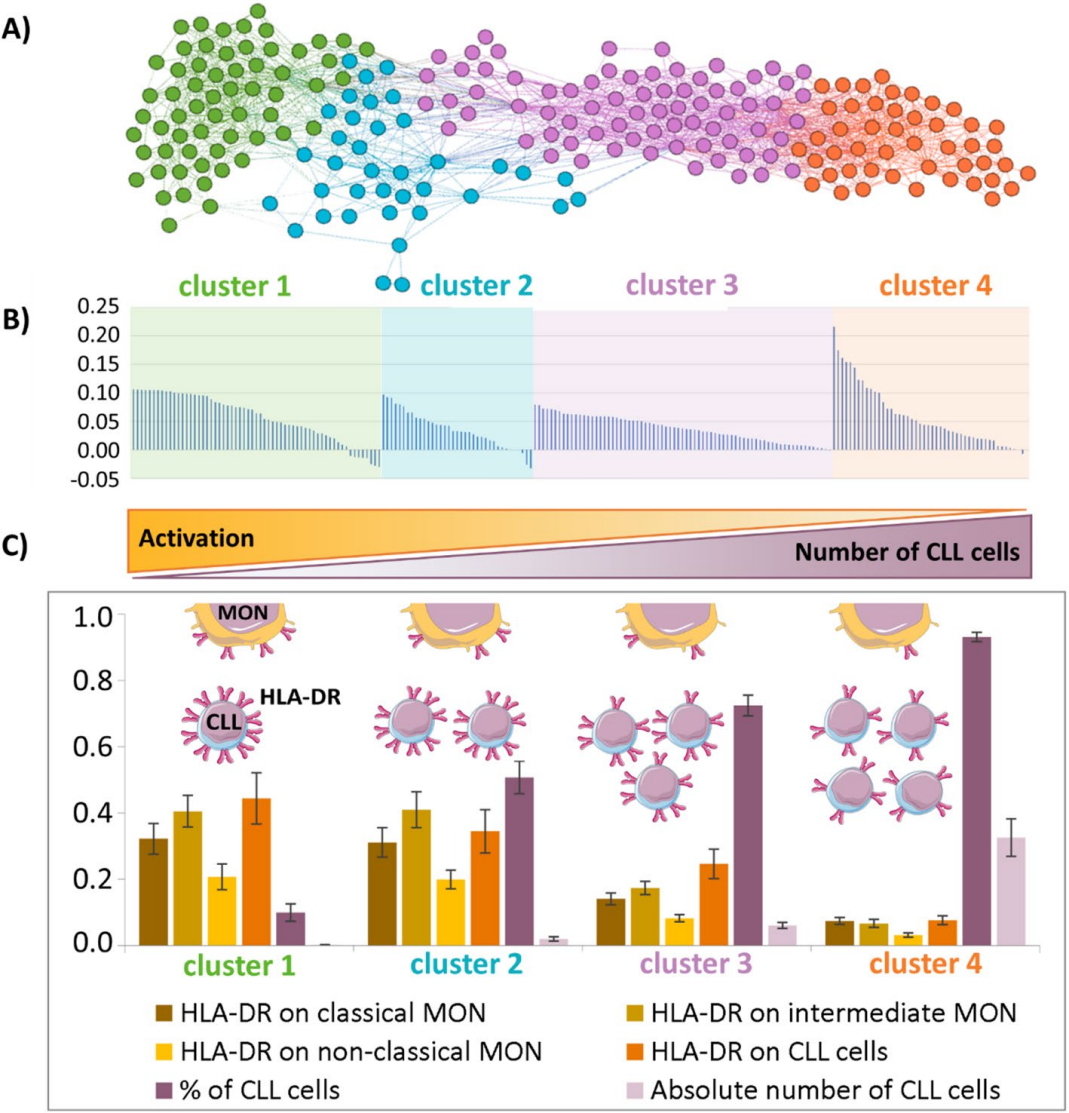
Patient Similarity Network (PSN) analysis of CLL patients based on the CLL cell bulk and activation of immune cells. (**A**) PSN shows the distribution of CLL patients into four clusters, according to percentage and CLL cell counts and the expression of HLA-DR on CLL cells and subsets of monocytes (classical, intermediate, non-classical MON). Individual clusters are coloured. Each node corresponds to one patient. Lines connect patients with the highest similarity of expression/cell count profiles. (**B**) The silhouette of clustered patients in the acquired PSN shows high positive values, indicating that the patients were unambiguously assigned to a cluster far from the boundary between two adjacent clusters; the presence of negative values indicates patients who may also be included in another cluster. (**C**) Characteristics of obtained clusters revealed by PSN. The y-axis in the graph shows the average values of the used markers/cell counts normalised to the maximum value in the data set. The whiskers represent confidence intervals.

PSNs were used to cluster patients with similar profiles, regarding the CLL counts and the activation status of immune cells. Of the investigated cells (CD4+ and CD8+ T lymphocytes, Tregs, NK cells, neutrophils, classical/intermediate/non-classical monocytes and CLL cells) and their activation markers, the best clustering of patients was observed using CLL counts and HLA-DR expression on CLL cells and classical, intermediate and non-classical monocyte subsets. As shown in Fig. 2A, CLL patients were clustered into four groups (clusters) showing the inverse relationship between the CLL cell counts and the activation of CLL and monocyte subsets. The silhouette showed that most patients were correctly assigned to the particular clusters (Fig. 2B). Generally, high CLL cell counts were associated with low activation status on CLL cells and all monocyte subsets, and conversely, low CLL cell counts with high activation of CLL cells, and classical, intermediate, and non-classical monocyte subsets (Fig. 2C).

### PSN analysis based on the activation markers of circulating immune cell microenvironment in CLL patients

In order to investigate the association of activation of immune cells with clinical parameters, we created PSN by combining activation markers of immune subpopulations, not taking into account the CLL cell counts.

**Figure 3:**
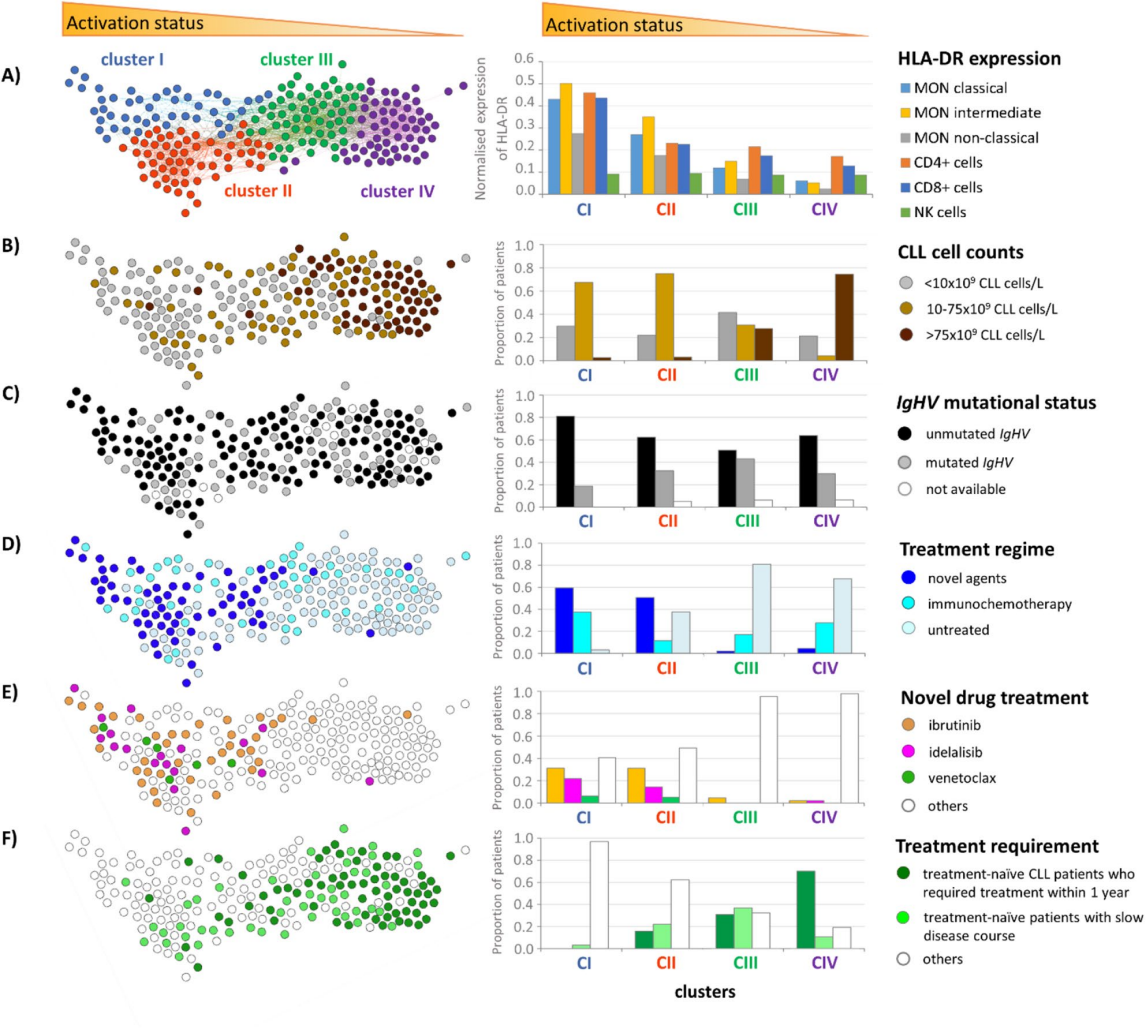
Patient Similarity Network (PSN) based on a combination of HLA-DR expression on monocyte subsets, CD4+ and CD8+ lymphocytes and NK cells and association of clinical parameters in particular clusters. The dots (= patients) were coloured based on the clinical data of each particular patient. Bar charts show the distribution of expression of the studied markers in individual clusters. (**A**) PSN consists of four clusters. Distribution of CLL patients and their proportion in particular clusters are visualised according to (**B**) CLL cell counts, (**C**) *IgHV* mutational status, (**D**) treatment regimen, (**E**) novel drug treatment, and (**F**) treatment requirement in treatment-naïve patients.

PSNs revealed four patient clusters based on similarities of the HLA-DR expression on subsets of monocytes (classical, intermediate, non-classical), CD4+ and CD8+ lymphocytes and NK cells (Fig. 3A). The silhouette and detailed characteristics of obtained clusters are shown in the online supplement (Table S4, Fig. S1). Patients within clusters I and II showed overlap of activation marker profiles on studied populations. For better visualisation, the network vertices (= patients) were coloured based on the clinical data of each particular patient (Fig. 3B–F). The patients with the highest activation of immune cells were clustered into clusters I and II, which were associated with the lowest CLL cell counts (Fig. 3B). We did not observe any association of mutational status of *IgHV* status (Fig. 3C), Binet stage, β-2-microglobulin, *TP53* disruption, CD49d and CD38 (data not shown) with particular cluster(s), showing that these clinical parameters are not associated with activation of immune cells.

Next, patients on novel drugs, such as ibrutinib (an irreversible inhibitor of Bruton's tyrosine kinase, BCR inhibitor), idelalisib (an inhibitor of the δ isoform of phosphatidylinositol 3-kinase, BCR inhibitor) and venetoclax (BCL2 inhibitor), were compared with treatment-naïve patients as well as with patients previously treated with immunochemotherapy. CLL patients treated with novel agents were assigned to the clusters with high activation of immune cells, irrespective of the type of drug used (Fig. 3D,E). CLL patients with highly activated immune cells were predominantly treatment-naïve with slow disease course and low counts of CLL cells. Treatment-naïve patients who required treatment within the follow-up period of one year were associated with clusters of less activated immune cells (Fig. 3F). Higher activation of immune cells with novel drugs was also confirmed on a subset of patients with an equal number of CLL cells treated with novel drugs and previous immunochemotherapy. Increased HLA-DR expression on the intermediate (*P* = 0.012) and non-classical (*P* = 0.028) monocytes and a decreased percentage of non-classical monocytes (*P* = 0.046), classical monocytes (*P* = 0.038) and Treg cells (*P* < 0.001) were detected in patients treated with novel agents (online supplement, Tables S5 and S6).

**Figure 4:**
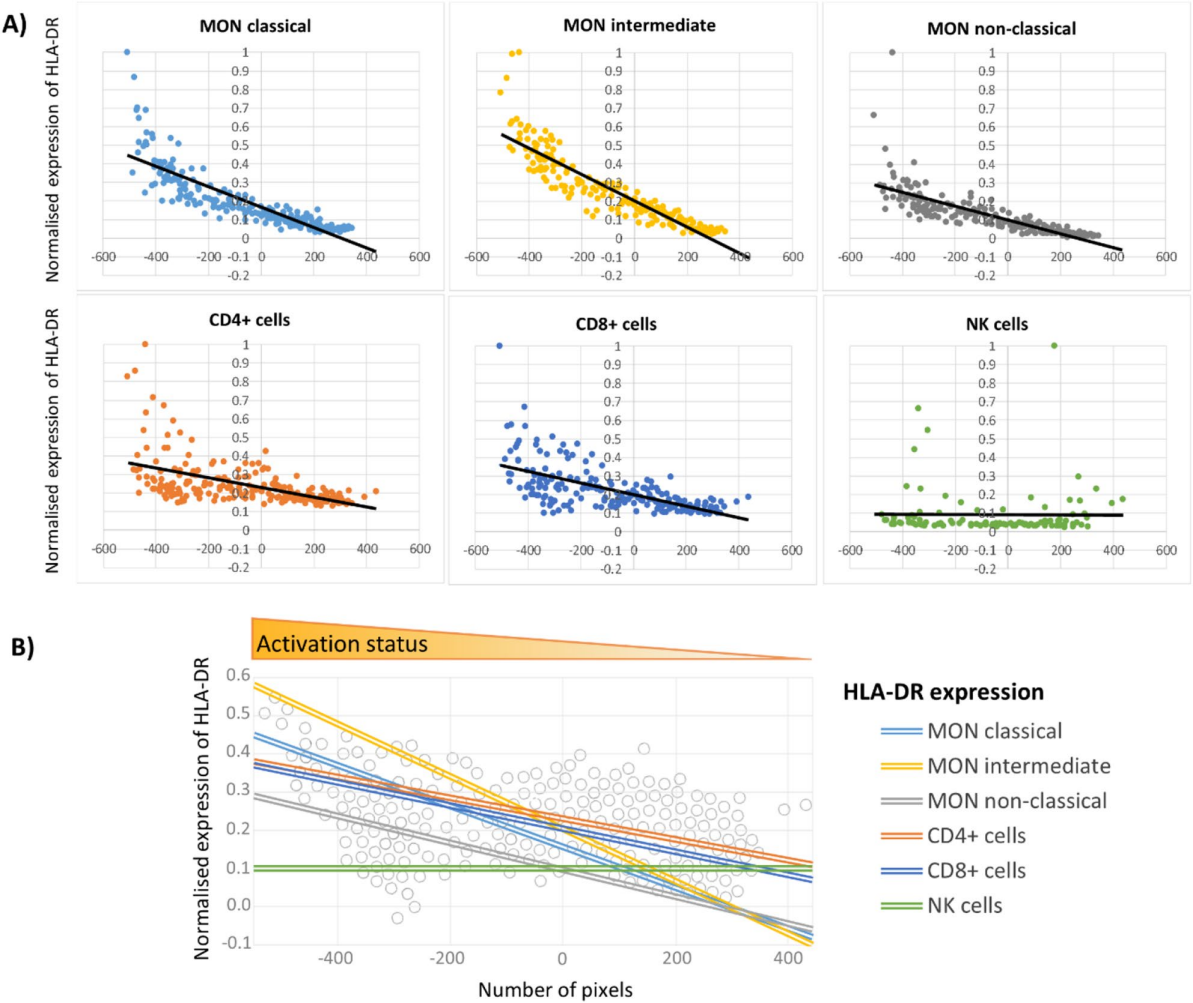
(**A**) Trend analysis of HLA-DR expression on monocyte (MON) subsets, CD4+ lymphocytes, CD8+ lymphocytes, and NK cells in PSN layout (presented in Fig. 3A). The y-coordinate of each dot in a scatterplot represents the expression value of studied markers in an individual patient. Values of all parameters were normalised to a maximum of 1 (the highest value in the data set). (**B**) Comprehensive visualisation of HLA-DR expression trends throughout the whole PSN. The unit on the x-axis is a pixel. The lines show the linear trend of the investigated parameter(s) obtained from scatterplots data.

Next, we calculated the trends of changes in the activation of immune cells across particular clusters. The obtained scatterplots expressed the relationships between the investigated activation markers and the horizontal position of the patient in the layout (Fig. 4A). Linear trends obtained from the scatterplots data for HLA-DR on all studied immune subpopulations except NK cells (Fig. 4B), emphasise the relationship between the patient's position in the PSN layout and the activation status and other clinical parameters.

### Time-dependent changes of HLA-DR on monocytes during long-term treatment with ibrutinib and idelalisib

We were also interested in whether the activation of immune cells differed in CLL patients treated with ibrutinib, idelalisib and venetoclax. Furthermore, we investigated the time-dependent dynamics of changes in the activation of immune cells of CLL patients on treatment with two BCR inhibitors: ibrutinib and idelalisib.

First, we analysed the expression of surface markers (CD27, CD38, CD49d and HLA-DR) on CLL cells in patients treated with ibrutinib, idelalisib, and venetoclax and we revealed no difference in the analysed markers between these patient groups. Of the immune cells studied, the percentage of intermediate monocytes was increased in patients treated with idelalisib comparing to ibrutinib and venetoclax (ibrutinib vs idelalisib: *P* = 0.002; idelalisib vs venetoclax: *P* = 0.006, Table S7). Moreover, patients treated with idelalisib showed high expression of HLA-DR (ibrutinib vs idelalisib: *P* = 0.007) and CD64 (ibrutinib vs idelalisib: *P* = 0.018; idelalisib vs venetoclax: *P* = 0.046) comparing to ibrutinib and venetoclax, irrespective of treatment duration.

**Figure 5:**
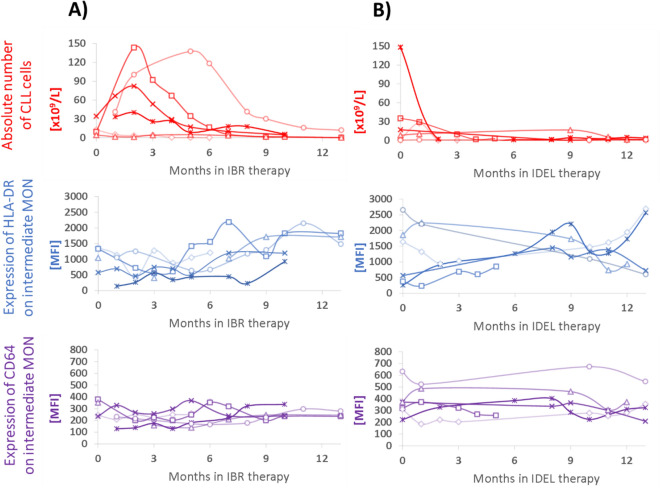
Time-dependent changes of CLL counts and expression of HLA-DR and CD64 on intermediate monocytes in CLL patients treated with (**A**) ibrutinib (IBR, n = 6) and (**B**) idelalisib (IDEL, n = 6). CLL cell count is shown using red dots and curves; the expression of HLA-DR and CD64 on intermediate monocytes using blue and violet graphs, respectively. Not all patients were sampled for each time point.

Analysis of longitudinal samples from patients treated with ibrutinib (n = 6) and idelalisib (n = 6) showed changes in the activation markers on immune cells, as well as the number of CLL cells, during the course of treatment, with marked differences between ibrutinib and idelalisib. The increase in the CLL cell counts within two months of initiating ibrutinib treatment was observed in the majority of patients, as also shown by others due to drug-induced redistribution of CLL cells between lymph nodes and circulation^25,26^, followed by a decline to almost zero values during the first year of treatment (Fig. 5A). Simultaneously, along with the decrease in the CLL count, the trend of increasing HLA-DR expression on classical and intermediate monocytes (Fig. 5A), as well as the non-classical monocytes and CD4+ and CD8+ T cells (data not shown), was detected during the first year of ibrutinib treatment, but not with idelalisib. CD64 expression on intermediate monocytes remained on the stable level (Fig. 5A). In patients treated with idelalisib, there was an evident decrease or stable level of CLL cell numbers during the first few months of treatment. However, compared to patients receiving ibrutinib treatment, the expression of HLA-DR and CD64 on monocytes had both a declining and an upward trend (Fig. 5B).

### HLA-DR expression on monocyte subpopulations does not differ between treatment-naïve CLL patients and healthy controls

Next, we analysed the differences in the activation of CLL and immune cells in treatment-naïve patients who required treatment (n = 61) within one year of sampling and treatment-naïve patients with the slow course (n = 38). Furthermore, all studied parameters were compared with those on CD19 + B cells from age- and sex-matched healthy subjects.

**Figure 6:**
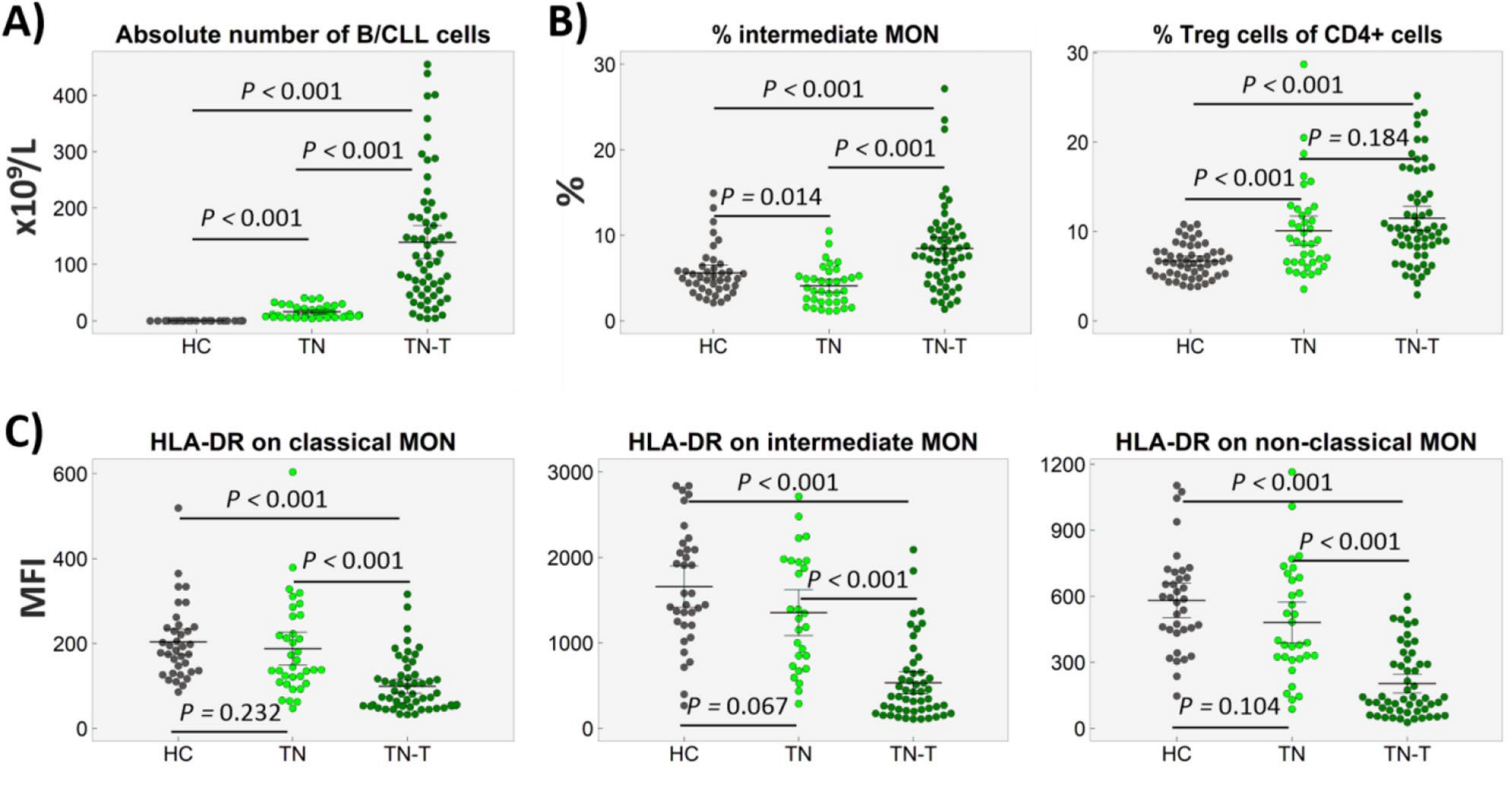
Distribution of circulating cells in treatment-naïve CLL patients who required treatment within one year (TN-T), treatment-naïve patients with the slow disease course (TN) and healthy controls (HC). (**A**) CLL cell counts in CLL patients or B cells in healthy controls. (**B**) Percentages of intermediate monocytes and proportion of Treg cells within CD4+ T cells. (**C**) Expression of HLA-DR on classical, intermediate and non-classical MON. In a minority of patients, the data are missing due to low abundance of monocyte cell subpopulations.

Treatment-naïve patients who required treatment during follow-up exhibited higher percentages and numbers of CLL cells (both *P* < 0.001) and an elevated percentage of intermediate monocytes (*P* < 0.001), compared to treatment-naïve patients with the slow course (median follow-up at 26 months). When comparing treatment-naïve CLL patients with healthy controls, the treatment-naïve patients with slow disease course did not differ from the healthy control subjects in terms of the activation of monocyte subsets (Fig. 6). The treatment-naïve patients requiring treatment within the one-year follow-up period had a lower activation on all monocyte subsets when compared to the healthy control patients. In all treatment-naïve patients, a higher proportion of Treg cells (*P* < 0.001) was detected in comparison to the healthy control patients (Fig. 6B).

### Percentage of intermediate monocytes is associated with time-to-treatment

**Figure 7:**
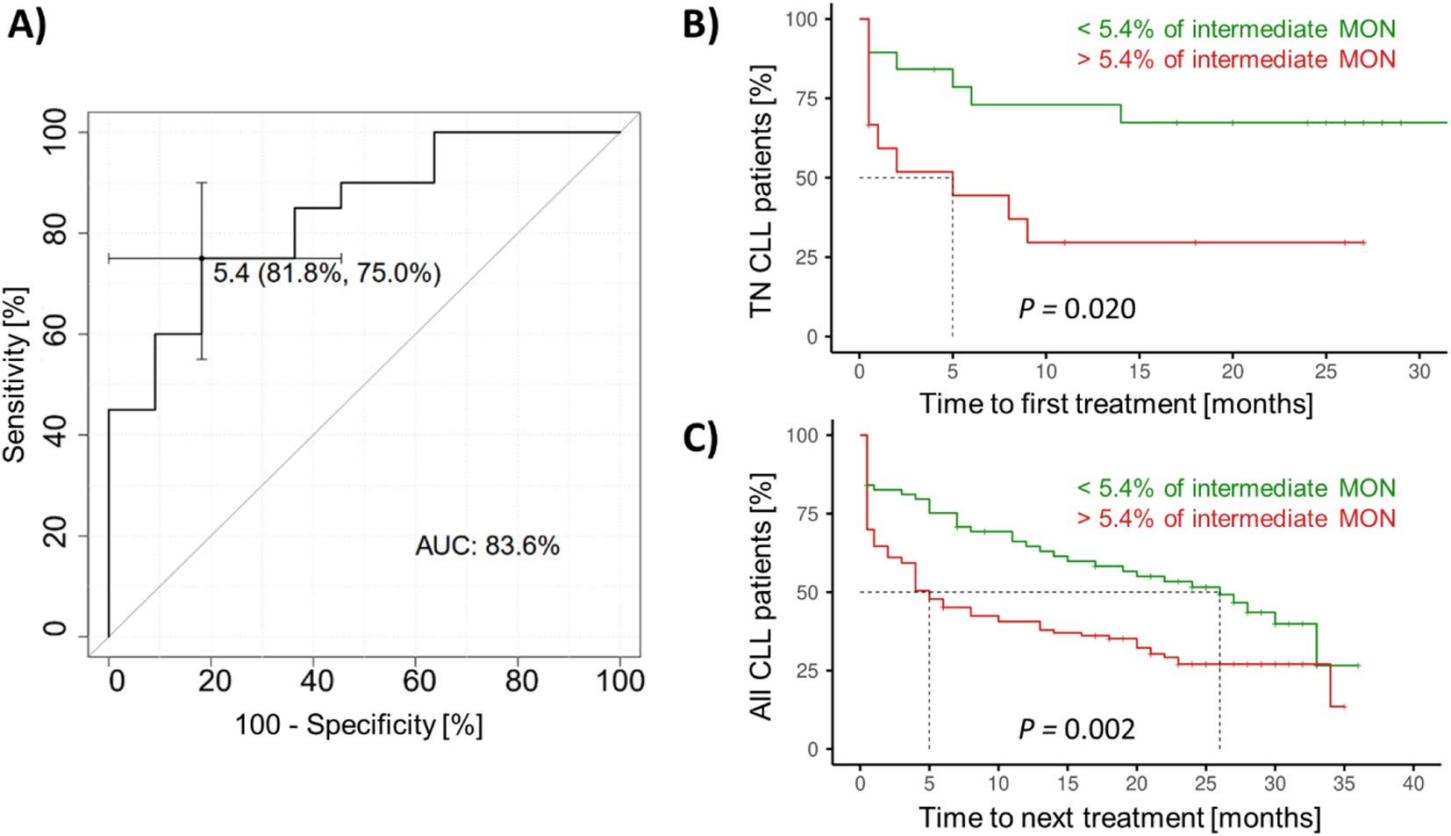
Predictive potential of intermediate monocyte percentage in CLL patients. (**A**) ROC curve for treatment-naïve patients sampled at the diagnosis, subdivided according to the treatment requirement during the post-sampling follow-up. K–M curves show time-to-treatment (**B**) in groups of newly diagnosed treatment-naïve (TN) CLL patients and (**C**) validation cohort of CLL patients in all stages of the disease. The green curves show patients with a low percentage of intermediate MON, while the red curves show patients with more than 5.4% of intermediate MON.

To estimate the association of circulating immune cells and/or their activation with TTT, we performed ROC analysis in newly diagnosed patients (n = 34, derivation cohort), subdivided according to the treatment requirement during one-year post-sampling follow-up. Of the studied markers and cell counts, the percentage of intermediate monocytes (cut-off 5.4%) achieved the most discriminatory power, reaching the area under the ROC curve (AUC) of 0.836 (Fig. 7A). Similar results were obtained when analysing newly diagnosed treatment-naïve patients, subdivided according to the low (< 4 points) vs high (≥ 4 points) CLL-IPI prognostic score (AUC = 0.805, data not shown), which includes age, Binet stage, level of serum β2-microglobulin, *IgHV* status, and *TP53* status^27^. Moreover, patients with a low percentage (< 5.4%) of intermediate monocytes had a higher expression of HLA-DR than those with a high percentage of intermediate monocytes (*P* = 0.020, online supplement, Fig. S2). Regarding the activation markers, HLA-DR expression on intermediate monocytes (AUC = 0.814) and classical monocytes (AUC = 0.764) also showed high discriminatory power for TTT, even when corrected for *IgHV* status, *TP53* disruption, CD38 and CD49d expressions, respectively.

Furthermore, Kaplan–Meier (K–M) curves were generated in CLL patient subgroups, subdivided according to the percentage of intermediate monocytes (cut-off 5.4%) in the derivation (n = 31) and validation cohorts (n = 170; 26 patients who died before starting treatment were excluded from this analysis), respectively. The K-M curves showed that CLL patients with more than 5.4% of intermediate monocytes had a shorter TTT than those with a lower percentage of intermediate monocytes in both derivation (*P* = 0.020, Fig. 7B) and validation cohorts (*P* = 0.002, Fig. 7C), respectively. Odds ratio calculation demonstrated that CLL patients with higher intermediate monocytes (> 5.4%), which were predominantly low-activated, were 2.5 times more likely (95% confidence interval 1.421–4.403, *P* = 0.002) to require treatment during the one-year follow-up than those with a low percentage of intermediate monocytes.

## Discussion

This first study on the complex characterisation of the blood microenvironment in CLL showed that activation of circulating monocytes and T cells is associated with CLL cell counts and the therapy used, with the lowest activation seen in patients with a progressive form of the disease and the highest activation in patients with slow disease course, untreated disease and those treated with novel agents (ibrutinib, idelalisib and venetoclax).

The dependency of CLL cells on signals from the microenvironment in the bone marrow and lymph nodes is widely accepted^2,3^, but the CLL cell cross-talk within the blood immune microenvironment is not yet fully characterised. We, therefore, investigated the blood immune cell microenvironment in terms of heterogeneity and the activation of immune cells and its relationship to the number of CLL cells. Of studied activation markers, our data revealed an unambiguous negative association for the bulk of CLL cells with the activation status of blood immune cells, particularly regarding the expression of HLA-DR. Moreover, the selection of this marker was supported by its association with the activation of all investigated immune cell populations and their subsets^13^. HLA-DR is considered to be a late activation marker on the surface of monocytes, macrophages, NK cells and B and T lymphocytes^28,29^. In the context of cancer biology, HLA-DR is reported to serve as a favourable prognostic marker for several types of cancer, such as colorectal carcinoma^30^, glioma^31^ and melanoma^32^. Moreover, HLA-DR expression is suggested as essential for establishing effective anti-tumor immunity via CD4+ cell activation and recruitment of effector cell populations such as macrophages, as shown in the experimental setting in myeloma^33^. In our patients, an increased number of CLL cells was associated with decreased HLA-DR expression by monocyte subsets, T cells, and CLL cells themselves. In contrast, a lower number of CLL cells was associated with the elevation of HLA-DR on CLL cells, as well as almost all immune cell subsets in circulation, reaching levels of the healthy control group.

In addition, HLA-DR expression on immune cells is proven to reflect the tumour immune status and the response to the therapy being implemented^34^. In line with the above evidence, we suggested that HLA-DR expression may also reflect disease progression and treatment response in CLL. To support this hypothesis, the low activation of monocyte subpopulations was observed in a group of untreated patients, requiring treatment during the one-year follow-up period. Conversely, patients with higher activation of monocyte subpopulations, particularly intermediate monocytes, were treatment-naïve patients with a slow disease course. The observed crucial impact of CLL bulk on the blood microenvironment is supported by a recent study showing that CLL cells impair mitochondrial fitness in CD8 + T cells and impede CAR T-cell efficacy^35^. Moreover, other studies have demonstrated that CLL cells are active participants in microenvironmental cross-talk^3^, contributing to the inhibition of effective immune cell activation through the production of a spectrum of immunosuppressive mediators^12,36,37^, most prominently in the lymph nodes^36^. Here, we also provide evidence of the immunosuppressive condition in the circulation of the patients with progressive disease course. The exact mechanism by which CLL suppresses the activation of bystander immune cells needs to be investigated in future studies.

Next, we explored whether circulation microenvironment may be of predictive value in CLL. Our analysis revealed that the percentage of intermediate and classical monocytes and their activation have the predictive potential for the TTT. In particular, a high percentage of intermediate monocytes (> 5.4%) with low activation was predictive for a progressive form of the disease, and these patients are 2.5 times more likely to require treatment than those with a low percentage of intermediate monocytes, irrespective of the treatment. The importance of monocytes for the prognosis of CLL patients has been demonstrated in recent studies, showing an association between monocyte counts, particularly of circulating classical monocytes, and time until the first therapy, aggressiveness and disease outcome^38,39^. Regarding monocytes and monocyte-derived cells in CLL, there is existing evidence that they promote CLL cell survival and proliferation^39^ and have deregulated genes involved in phagocytosis and inflammation^12^. The key role of monocytes in CLL is also supported by observations in animal models of CLL, where the depletion of monocytes and macrophages resulted in control of CLL development and reparation of immune dysfunction in vivo^40^. The exact mechanisms by which monocytes, particularly intermediate monocytes exhibiting potent pro-inflammatory properties upon stimulation^41,42^, contribute to the pathogenesis of CLL has not been fully clarified. There is first evidence about in vitro differentiation of blood mononuclear CD14+ cells in the context of CLL into nurse-like cells (NLCs) that can protect CLL cells from apoptosis^43,44^. The link between intermediate monocytes and NLCs is further supported by the expression of the angiopoietin receptor Tie-2 on both cell types, thus contributing to the tumour-promoting environment in CLL^12,45^. In solid tumours, the circulating intermediate monocytes differentiate into the tumour-associated macrophages^43,44^.

Despite our study demonstrating the dependency of the activation of blood bystander cells on the bulk of CLL cells, no correlation was detected with other prognostic markers for CLL, such as *IgHV* status, Binet stage, β-2-microglobulin, *TP53* disruption, CD49d and CD38. It seems that these prognostic markers do not have a direct impact on the establishment of the immune microenvironment but do have an influence on CLL cell counts and, correspondingly, on the disease course, survival time and risk-adapted treatment options, as shown in other studies^7,46,47^. In addition to the emerging concepts of genetic makeup, our data highlights the CLL cells as an additional determinant of the immune contexture in CLL. The need to clarify the causal link between genetic landscape, immunophenotype and cytokines/chemokines produced by CLL cells and bystander immune cells (and their function and behaviour), factors influencing the course of the disease, as well as the results of intervention strategies, remains.

Following this, we turned our attention to changes in the blood microenvironment of patients treated with ibrutinib, idelalisib and venetoclax, all of which have recently emerged as highly promising, novel, therapeutic strategies in CLL^5–7^. Although these drugs predominantly target CLL cells^5–7,48^, there is growing evidence of their effects on the bystander immune cells. Ibrutinib and idelalisib have been proven to have an immunomodulatory effect on T cells^5,13,49–52^, NK cells^53^, monocytes^13,54^, and the production of Th2 cytokines^55^, depending on the time of treatment^13^. Venetoclax has been shown to modulate the number and activation of T and B cells, restore NK cell function and reduce the overproduction of inflammatory cytokines^36^. In our study, multivariate analyses revealed the activation of all circulating immune cells in the majority of patients treated with novel drugs. More importantly, the activation of all immune cells in the majority of our patients receiving novel drugs was more pronounced, compared with those previously treated with immunochemotherapy or untreated patients, even after correction for CLL cell number. In line with our data, a recent study using venetoclax-based regimens revealed a reduction in the immunosuppressive footprint of CLL, suggesting that immune system regeneration occurs after the removal of leukemic cells^36^. Similarly, ibrutinib has been shown to modulate the immunosuppressive CLL microenvironment, through the STAT3-mediated suppression of regulatory B cell function and inhibition of the PD-1/PD-L1 pathway^56^. Our data further support the key contribution of novel agents to the immunomodulation of the circulating microenvironment in CLL. The exact mechanism of action of the novel agents on the microenvironment still needs to be clarified, while both an indirect effect—due to the elimination of neoplastic cells—and direct activation of immune cells are expected. Interestingly, some of the patients on novel agents had a notably low HLA-DR expression on their immune cells. Since HLA-DR expression on immune cells may reflect a response to therapy, as demonstrated in breast cancer patients^34^, our patients on novel drugs with low immune cell activation may be at high risk of the disease progression. Interestingly, our data showed different time-dependent dynamics of immune cell activation and redistribution of CLL cells from tissues to peripheral blood when treated with ibrutinib and idelalisib, respectively. Our data highlights the critical effect of treatment regimens for both drugs not only on the abrogation of BCR-dependent survival and proliferation signals but also on the activation of immune cells and establishing the functionality of non-tumour cells.

This study has some limitations. First, we analysed a real-world cohort of CLL patients, sampled at different time points and treatment regimens, and only several patients had longitudinal samples. Second, we did not have paired lymph node or bone marrow samples to examine these microenvironments simultaneously. Third, we did not perform a detailed study of the mechanisms underlying the associations between CLL cells and activation of immune cells and characterisation of the underlying immune response. Despite these limitations, we believe that this initial study on deciphering the complex circulating microenvironment through advanced multivariate analysis highlights the vital contribution of immune cells to the outcome and treatment response of CLL.

## Conclusions

In summary, this study provides evidence demonstrating that the shaping of peripheral blood immune cells is strongly associated with the bulk of CLL cells. Our findings highlight the role of monocytes, particularly intermediate monocytes, in the pathogenesis of CLL, and they also nominate them as an independent predictive marker for time-to-treatment. Assessment of responsiveness to the therapy based on the profile of immune cells may represent a next-step in improving the success of current immunotherapies and the development of next-generation novel drugs.

## Supplementary Information

Supplementary Information.

## Data Availability

The data and materials of this study are available from the corresponding author on reasonable request.

## Abbreviations

AUC: Area under the ROC curve
Bcl-2: B cell lymphoma 2
BCR: B cell receptor
CLL: Chronic lymphocytic leukaemia
CLL-IPI: CLL-International prognostic index
FSC-A/-H: Forward scatter-area/-height
HC: Healthy controls
*IgHV*: Immunoglobulin heavy chain variable region
K–M curve: Kaplan–Meier curve
MFI: Median fluorescence intensity
MON: Monocytes
n.a.: Not available
OR: Odds ratio
PSN: Patient similarity network
ROC: Receiver operating curves
TN: Treatment-naïve
TN-T: Treatment-naïve CLL patients who required treatment
TTT: Time-to-treatment

## References

[1] Zhang S, Kipps TJ (2014). The pathogenesis of chronic lymphocytic leukemia. Annu. Rev. Pathol..

[2] Ten Hacken E, Burger JA (2016). Microenvironment interactions and B-cell receptor signaling in Chronic Lymphocytic Leukemia: implications for disease pathogenesis and treatment. Biochim. Biophys. Acta..

[3] van Attekum MH, Eldering E, Kater AP (2017). Chronic lymphocytic leukemia cells are active participants in microenvironmental cross-talk. Haematologica.

[4] Herishanu Y (2011). The lymph node microenvironment promotes B-cell receptor signaling, NF-kappaB activation, and tumor proliferation in chronic lymphocytic leukemia. Blood.

[5] Niemann CU (2016). Disruption of in vivo chronic lymphocytic leukemia tumor-microenvironment interactions by ibrutinib: findings from an investigator-initiated phase II study. Clin. Cancer Res..

[6] Schiattone L, Ghia P, Scarfò L (2019). The evolving treatment landscape of chronic lymphocytic leukemia. Curr. Opin. Oncol..

[7] Hallek M (2019). Chronic lymphocytic leukemia: 2020 update on diagnosis, risk stratification and treatment. Am. J. Hematol..

[8] Riches JC (2013). T cells from CLL patients exhibit features of T-cell exhaustion but retain capacity for cytokine production. Blood.

[9] Palma M (2017). T cells in chronic lymphocytic leukemia display dysregulated expression of immune checkpoints and activation markers. Haematologica.

[10] Manukyan G (2017). Neutrophils in chronic lymphocytic leukemia are permanently activated and have functional defects. Oncotarget..

[11] MacFarlane AW (2017). NK cell dysfunction in chronic lymphocytic leukemia is associated with loss of the mature cells expressing inhibitory killer cell Ig-like receptors. Oncoimmunology..

[12] Maffei R (2013). The monocytic population in chronic lymphocytic leukemia shows altered composition and deregulation of genes involved in phagocytosis and inflammation. Haematologica.

[13] Manukyan G (2018). Dynamic changes in HLA-DR expression during short-term and long-term ibrutinib treatment in patients with chronic lymphocytic leukemia. Leuk. Res..

[14] Hallek M (2008). Guidelines for the diagnosis and treatment of chronic lymphocytic leukemia: a report from the international workshop on chronic lymphocytic leukemia updating the national cancer institute-working group 1996 guidelines. Blood.

[15] Del Giudice I (2019). Minimal residual disease in chronic lymphocytic leukemia: a new goal?. Front. Oncol..

[16] Petrackova A (2019). Standardization of sequencing coverage depth in NGS: recommendation for detection of clonal and subclonal mutations in cancer diagnostics. Front. Oncol..

[17] Obr A (2018). TP53 mutation and complex karyotype portends a dismal prognosis in patients with mantle cell lymphoma. Clin. Lymphoma. Myeloma. Leuk..

[18] Kruzova L (2019). Complex karyotype as a predictor of high-risk chronic lymphocytic leukemia: a single center experience over 12 years. Leuk. Res..

[19] Diks AM (2019). Impact of blood storage and sample handling on quality of high dimensional flow cytometric data in multicenter clinical research. J. Immunol. Methods..

[20] Rühle PF, Fietkau R, Gaipl US, Frey B (2016). Development of a modular assay for detailed immunophenotyping of peripheral human whole blood samples by multicolor flow cytometry. Int. J. Mol. Sci..

[21] Ochodkova E, Zehnalova S, Kudelka M, Cao Y, Chen J (2017). Graph construction based on local representativeness. Computing and Combinatorics.

[22] Kriegova E (2018). Gender-related differences observed among immune cells in synovial fluid in knee osteoarthritis. Osteoarthr. Cartil..

[23] Gallo J, Kriegova E, Kudelka M, Lostak J, Radvansky M (2020). Gender differences in contribution of smoking, low physical activity, and high BMI to increased risk of early reoperation after TKA. J. Arthroplasty..

[24] Blondel VD, Guillaume JL, Lambiotte R, Lefebvre E (2008). Fast unfolding of communities in large networks. J. Stat. Mech. Theory Exp..

[25] Herman SEM (2014). Ibrutinib-induced lymphocytosis in patients with chronic lymphocytic leukemia: correlative analyses from a phase II study. Leukemia.

[26] Woyach JA (2014). Prolonged lymphocytosis during ibrutinib therapy is associated with distinct molecular characteristics and does not indicate a suboptimal response to therapy. Blood.

[27] International CLL-IPI working group (2016). An international prognostic index for patients with chronic lymphocytic leukaemia (CLL-IPI): a meta-analysis of individual patient data. Lancet Oncol..

[28] Reddy M, Eirikis E, Davis C, Davis HM, Prabhakar U (2004). Comparative analysis of lymphocyte activation marker expression and cytokine secretion profile in stimulated human peripheral blood mononuclear cell cultures: an in vitro model to monitor cellular immune function. J. Immunol. Methods..

[29] Saez-Cirion A (2007). HIV controllers exhibit potent CD8 T cell capacity to suppress HIV infection ex vivo and peculiar cytotoxic T lymphocyte activation phenotype. Proc. Natl. Acad. Sci. USA.

[30] Sconocchia G (2014). HLA class II antigen expression in colorectal carcinoma tumors as a favorable prognostic marker. Neoplasia..

[31] Diao J (2015). Overexpression of HLA-DR is associated with prognosis of glioma patients. Int. J. Clin. Exp. Pathol..

[32] Johnson DB (2016). Melanoma-specific MHC-II expression represents a tumour-autonomous phenotype and predicts response to anti-PD-1/PD-L1 therapy. Nat. Commun..

[33] Corthay A (2005). Primary antitumor immune response mediated by CD4+ T cells. Immunity.

[34] Saraiva DP, Jacinto A, Borralho P, Braga S, Cabral MG (2018). HLA-DR in cytotoxic T lymphocytes predicts breast cancer patients' response to neoadjuvant chemotherapy. Front. Immunol..

[35] van Bruggen JAC (2019). Chronic lymphocytic leukemia cells impair mitochondrial fitness in CD8+ T cells and impede CAR T-cell efficacy. Blood.

[36] de Weerdt I (2019). Distinct immune composition in lymph node and peripheral blood of CLL patients is reshaped during venetoclax treatment. Blood Adv..

[37] Arruga F (2020). Immune response dysfunction in chronic lymphocytic leukemia: dissecting molecular mechanisms and microenvironmental conditions. Int. J. Mol. Sci..

[38] Lapuc I (2015). Circulating classical CD14++CD16- monocytes predict shorter time to initial treatment in chronic lymphocytic leukemia patients: Differential effects of immune chemotherapy on monocyte-related membrane and soluble forms of CD163. Oncol. Rep..

[39] Friedman DR (2016). Relationship of blood monocytes with chronic lymphocytic leukemia aggressiveness and outcomes: a multi-institutional study. Am. J. Hematol..

[40] Hanna BS (2016). Depletion of CLL-associated patrolling monocytes and macrophages controls disease development and repairs immune dysfunction in vivo. Leukemia.

[41] Stansfield BK, Ingram DA (2015). Clinical significance of monocyte heterogeneity. Clin. Transl. Med..

[42] Merah-Mourah F, Cohen SO, Charron D, Mooney N, Haziot A (2020). Identification of novel human monocyte subsets and evidence for phenotypic groups defined by interindividual variations of expression of adhesion molecules. Sci. Rep..

[43] Tsukada N, Burger JA, Zvaifler NJ, Kipps TJ (2002). Distinctive features of "nurselike" cells that differentiate in the context of chronic lymphocytic leukemia. Blood.

[44] Boissard F (2016). Nurse-like cells promote CLL survival through LFA-3/CD2 interactions. Oncotarget..

[45] Coffelt SB (2011). Angiopoietin 2 stimulates TIE2-expressing monocytes to suppress T cell activation and to promote regulatory T cell expansion. J. Immunol..

[46] Eichhorst B, Hallek M (2016). Prognostication of chronic lymphocytic leukemia in the era of new agents. Hematol. Am. Soc. Hematol. Educ. Program..

[47] Boddu P, Ferrajoli A (2018). Prognostic factors in the era of targeted therapies in CLL. Curr. Hematol. Malig. Rep..

[48] Sedlarikova L, Petrackova A, Papajik T, Turcsanyi P, Kriegova E (2020). Resistance-associated mutations in chronic lymphocytic leukaemia patients treated with novel agents. Front. Oncol..

[49] Dubovsky JA (2013). Ibrutinib is an irreversible molecular inhibitor of ITK driving a Th1-selective pressure in T lymphocytes. Blood.

[50] Martinelli S (2018). Idelalisib impairs T-cell-mediated immunity in chronic lymphocytic leukemia. Haematologica.

[51] Parry HM (2019). Long-term ibrutinib therapy reverses CD8+ T cell exhaustion in B cell chronic lymphocytic leukaemia. Front. Immunol..

[52] Long M (2017). Ibrutinib treatment improves T cell number and function in CLL patients. J. Clin. Invest..

[53] Kohrt HE (2014). Ibrutinib antagonises rituximab-dependent NK cell-mediated cytotoxicity. Blood.

[54] Fiorcari S (2016). Ibrutinib modifies the function of monocyte/macrophage population in chronic lymphocytic leukemia. Oncotarget..

[55] Pleyer C, Wiestner A, Sun C (2018). Immunological changes with kinase inhibitor therapy for chronic lymphocytic leukemia. Leuk. Lymphoma..

[56] Kondo K (2018). Ibrutinib modulates the immunosuppressive CLL microenvironment through STAT3-mediated suppression of regulatory B-cell function and inhibition of the PD-1/PD-L1 pathway. Leukemia.

