# Supplementary material for: Deciphering the complex circulating immune cell microenvironment in chronic lymphocytic leukaemia using patient similarity networks
Source: Sci Rep. 2021 Jan 11;11:322. doi: 10.1038/s41598-020-79121-4 (PMC7801466; doi:10.1038/s41598-020-79121-4)
Supplement: Supplementary file 1 — Supplementary Information. [file 41598_2020_79121_MOESM1_ESM.pdf]

Supplementary Data

Deciphering the complex circulating immune cell microenvironment in chronic lymphocytic leukaemia using patient similarity networks

Zuzana Mikulkova, Gayane Manukyan, Peter Turcsanyi, Milos Kudelka, Renata Urbanova, Jakub Savara, Eliska Ochodkova, Yvona Brychtova, Jan Molinsky, Martin Simkovic, David Starostka, Jan Novak, Ondrej Janca, Martin Dihel, Pavlina Ryznerova, Lekaa Mohammad, Tomas Papajik & Eva Kriegova.

CONTENTS

Tables:

Table S1. Combinations of surface and activation markers used to characterise the immune populations.

Table S2. Description of test tubes of the panel with used surface markers, clones and fluorochromes (conjugates).

Table S3. Correlations between absolute numbers of chronic lymphocytic leukaemia (CLL) cells and expression of activation markers on immune cell populations or percentages of immune cell subpopulations.

Table S4. Characteristics of clusters detected in PSN (presented in Figure 3A in the main manuscript) based on immune cell activation markers in CLL patients.

Tables S5. Comparison of immune subset percentages, cell counts and activation markers expressed on immune cells in untreated CLL patients and CLL patients after the immunochemotherapy treatment with the same CLL cell counts (20–80x10^9/L).

Table S6. Comparison of immune subset percentages and activation markers expressed on immune cells in CLL patients after the immunochemotherapy treatment and patients on novel therapy with the equal CLL cell counts (<10.0x10^9/L).

Table S7. Comparison of immune subset percentages and activation markers expressed on immune cells in CLL patients treated with ibrutinib, idelalisib and venetoclax.

Figures:

Figure S1. The silhouette of clusters detected in PSN (presented in Figure 3A in the main manuscript) based on immune cell activation markers in CLL patients.

Figure S2. The difference in HLA-DR expression on intermediate monocytes (MON) between CLL patients with lower and higher numbers of intermediate monocytes (cut-off 5.4%).

Text:

Regardless of CLL cell count, the activation of circulating immune cells is dependent on the treatment regimen.

Patient Similarity Network (PSN) analysis.

Use of network layout to analyse trends.

References

Table S1. Combinations of surface and activation markers used to characterise the immune populations.

**Immune population**	**Combination of the markers**
CLL cells	CD5/CD19/CD20/CD27/CD38/CD49d/HLA-DR
Classical/intermediate/non-classical monocytes	CD11b/CD14/CD16/CD64/HLA-DR
Neutrophils	CD11b/CD15/CD16/CD54/CD62L/CD64
CD4+ T lymphocytes	CD3/CD4/HLA-DR
CD8+ T lymphocytes	CD3/CD8/HLA-DR
Treg lymphocytes	CD3/CD4/CD25/CD127
NK cells	CD3/CD16/CD56/CD69/HLA-DR

Table S2. Description of test tubes of the panel with used surface markers, clones and fluorochromes (conjugates).

**Description of tubes**	**FITC (clone)**	**PE (clone)**	**PerCP/ Cy5.5 (clone)**	**PE-Cy7 (clone)**	**APC (clone)**	**APC-Cy7 (clone)**
**CLL cells (tube 1)**	CD27 (M-T271)	CD20 (2H7)	CD5 (UCHT2)	CD38 (HB-7)	HLA-DR (L243)	CD19 (SJ25C1)
**CLL cells (tube 2)**	CD27 (M-T271)		CD5 (UCHT2)	CD38 (HB-7)	CD49d (9F10)	CD19 (SJ25C1)
**T cells**	CD3 (OKT3)	CD25 (M-A251)	CD127 (A019D5)	CD8 (SK1)	HLA-DR (L243)	CD4 (RPA-T4)
**NK cells (tube 1)**	CD3 / (CD16+CD56) (UCHT1 / 3G8+MEM-188)		-	-	CD69 (FN50)	CD4 (RPA-T4)
**NK cells (tube 2)**	CD3 / (CD16+CD56) (UCHT1 / 3G8+MEM-188)		-	-	HLA-DR (L243)	CD4 (RPA-T4)
**Monocytes**	CD14 (HCD14)	CD16 (3G8)	CD11b (ICRF44)	-	HLA-DR (L243)	CD64 (10.1)
**Neutrophils**	CD54 (HA58)	CD16 (3G8)	CD11b (ICRF44)	CD15 (W6D3)	CD62L (DREG-56)	CD64 (10.1)

Table S3. Correlations between absolute numbers of CLL cells and expression of activation markers on immune cell populations or percentages of immune cell subpopulations.

**Studied populations and their markers**	**r_s value**	***P* value**	***Mean value (min-max)***
**Classical monocytes (MON)**			
% of MON	-0.05	0.480	82.0 (41.0–98.6)
HLA-DR MFI	-0.76	**< 0.001**	207 (33.4–854)
CD64 MFI	-0.39	**< 0.001**	284 (109–743)
CD11b MFI	-0.11	0.120	637 (151–1811)
**Intermediate MON**			
% of MON	-0.01	0.880	7.67 (1.15–24.2)
HLA-DR MFI	-0.74	**< 0.001**	1218 (109–4698)
CD64 MFI	-0.52	**< 0.001**	256 (105–649)

CD11b MFI	-0.07	0.330	538 (139–1165)
**Non-classical MON**			
% of MON	0.11	0.120	9.98 (0.03–41.1)
HLA-DR MFI	-0.78	**< 0.001**	540 (43.0–2868)
CD64 MFI	-0.13	0.086	153 (90.2–355)
CD11b MFI	0.18	**0.014**	108 (36.1–286)
**Neutrophils**			
CD64 MFI	-0.22	**0.036**	81.7 (33.7–163)
CD54 MFI	0.10	0.310	20.5 (14.7–54.2)
CD11b MFI	-0.08	0.400	159 (23.9–533)
CD62L MFI	-0.57	**< 0.001**	399 (53.8–1395)
**NK cells**			
HLA-DR MFI	-0.11	0.270	67.4 (17.3–723)
CD69 MFI	-0.33	**< 0.001**	32.7 (14.5–109)
**T cells**			
CD4+/CD8+ ratio	0.22	**< 0.001**	1.47 (0.17–5.63)
HLA-DR (MFI) on CD4+ cells	-0.51	**< 0.001**	24.4 (13.0–99.1)
HLA-DR (MFI) on CD8+ cells	-0.60	**< 0.001**	34.5 (15.1–158)
% **Treg cells** of CD4+ cells	0.31	**< 0.001**	9.80 (1.42–28.7)

MFI, mean fluorescence intensity.

Table S4. Characteristics of clusters detected in PSN (presented in Figure 3A in the main manuscript) based on immune cell activation markers in CLL patients. The expressions of the used markers were normalised to the maximum value in the data set.

**Normalised HLA-DR expression**	**Cluster I** *Mean (CI)*	**Cluster II** *Mean (CI)*	**Cluster III** *Mean (CI)*	**Cluster IV** *Mean (CI)*
**Classical MON**	0.43 (0.36–0.50)	0.27 (0.26–0.28)	0.12 (0.10–0.14)	0.06 (0.05–0.07)
**Intermediate MON**	0.50 (0.43–0.58)	0.35 (0.34–0.37)	0.15 (0.12–0.18)	0.05 (0.04–0.06)
**Non-classical MON**	0.27 (0.21–0.34)	0.17 (0.17–0.18)	0.07 (0.05–0.08)	0.02 (0.02–0.03)
**CD4+ cells**	0.46 (0.39–0.53)	0.23 (0.22–0.25)	0.21 (0.20–0.23)	0.17 (0.16–0.18)
**CD8+ cells**	0.44 (0.38–0.49)	0.23 (0.21–0.24)	0.17 (0.16–0.19)	0.13 (0.12–0.14)
**NK cells**	0.09 (0.04–0.15)	0.09 (0.02–0.17)	0.09 (0.04–0.13)	0.09 (0.06–0.12)

CI, confidence interval; MON, monocytes.

Regardless of CLL cell count, the activation of circulating immune cells is dependent on the treatment regimen

To recognise the differences in circulating cells between groups of patients with different treatment regimens, we compared studied parameters in patients with the comparable levels of CLL cells to reduce the impact of CLL cell number on studied parameters. We compared untreated patients (n=36) with treated patients with chemotherapy in the past (n=18) with CLL cell count from 20.0 to 80.0x10^9 CLL cells/L and previously treated patients with

immunochemotherapy (n=22) with patients treated with the novel drugs (n=53) with CLL cell count lower than $10.0x10^9$ CLL cells /L. The untreated patient group was not compared with the group of patients treated with novel drugs because the CLL cell number in both groups was mostly incomparable.

Comparison of treatment-naïve patients with patients after chemotherapy (Table S4) revealed higher activation of immune cells in patients after chemotherapy. Particularly, the expression of HLA-DR on CD4+ (P<0.001) and CD8+ lymphocytes (P=0.013), classical (P=0.032) and non-classical (P=0.030) subsets of monocytes, as well as expression of CD64 on classical monocytes (P=0.044) and CD11b on non-classical monocytes (P=0.041), were lower in the untreated group. CD4+/CD8+ ratio was found to be higher in untreated patients (P=0.008).

When we compared the patients with passed chemotherapy treatment with the patients on novel drug therapy, higher activation of immune cells was observed in patients treated with novel drugs (Table S5).

Table S5. Comparison of immune subset percentages, cell counts and activation markers expressed on immune cells in untreated CLL patients and CLL patients after the immunochemotherapy treatment with the same CLL cell counts ($20–80x10^9$/L).

**Populations/markers**	**Untreated** ***Mean (CI)***	**Chemotherapy** ***Mean (CI)***	***FC***	***P value***
**CLL cell count [x10^9/L]**	44.0 (37.5–50.6)	42.2 (32.3–51.9)	-0.07	0.683
**Classical monocytes (MON)**				
% of MON	85.3 (82.6–88.0)	82.6 (77.9–87.3)	-0.04	0.257
Cell count [x10^9/L]	0.46 (0.38–0.54)	0.45 (0.30–0.60)	-0.02	0.880
HLA-DR MFI	122 (101–142)	172 (130–215)	0.57	**0.032**
CD64 MFI	229 (209–249)	277 (221–333)	0.20	**0.044**
CD11b MFI	544 (462–625)	604 (513–694)	0.10	0.228
**Intermediate MON**				
% of MON	5.32 (4.23–6.41)	6.61 (5.14–8.08)	0.36	0.130
Cell count [x10^9/L]	0.03 (0.02–0.03)	0.03 (0.02–0.05)	0.31	0.103
HLA-DR MFI	792 (554–1031)	1034 (645–1423)	0.24	0.133
CD64 MFI	200 (174–227)	258 (191–326)	0.19	0.061
CD11b MFI	444 (373–515)	573 (485–661)	0.35	0.050
**Non-classical MON**				
% of MON	9.38 (7.36–11.4)	10.8 (6.32–15.2)	0.21	0.645
Cell count [x10^9/L]	0.04 (0.03–0.05)	0.05 (0.03–0.06)	0.17	0.250

HLA-DR MFI	285 (222–348)	519 (304–734)	0.38	**0.030**
CD64 MFI	146 (135–158)	170 (133–208)	0.01	0.615
CD11b MFI	89.9 (77.9–102)	116 (94.6–138)	0.19	**0.041**
**Neutrophils**				
Cell count [x10^9/L]	5.38 (4.33–6.43)	4.26 (3.47–5.05)	-0.21	0.335
CD64 MFI	69.5 (58.9–80.1)	80.9 (74.0–87.8)	0.04	0.162
CD54 MFI	20.4 (17.4–23.5)	19.0 (16.8–21.2)	-0.10	0.708
CD11b MFI	178 (124–232)	159 (123–196)	-0.18	0.615
CD62L MFI	275 (201–350)	306 (139–472)	0.03	0.965
**NK cells**				
Cell count [x10^9/L]	0.48 (0.33–0.63)	0.34 (0.24–0.43)	-0.30	0.254
HLA-DR MFI	83.8 (5.17–173)	42.7 (6.55–78.8)	-0.15	0.958
CD69 MFI	29.1 (23.5–34.7)	25.1 (21.9–28.3)	-0.02	0.792
**T cells**				
Cell count [x10^9/L]	2.89 (2.41–3.36)	2.75 (1.90–3.60)	-0.05	0.660
CD4+/CD8+ ratio	2.02 (1.57–2.47)	1.04 (0.75–1.34)	-0.31	**0.008**
HLA-DR (MFI) on CD4+ cells	19.2 (17.7–20.7)	27.8 (22.5–33.1)	0.25	**< 0.001**
HLA-DR (MFI) on CD8+ cells	25.2 (22.0–28.4)	36.1 (27.2–45.1)	0.12	**0.013**
**% Treg cells** of CD4+ cells	10.7 (9.19–12.2)	10.7 (8.39–13.0)	0.01	0.949
Cell count [x10^9/L]	0.14 (0.10–0.18)	0.09 (0.07–0.12)	-0.33	0.278

CI, confidence interval; MFI, mean fluorescence intensity.

Table S6. Comparison of immune subset percentages and activation markers expressed on immune cells in CLL patients after the immunochemotherapy treatment and patients on novel therapy with the equal CLL cell counts (<10.0x10^9/L).

**Populations/markers**	**Chemotherapy** ***Mean (CI)***	**Novel drugs** ***Mean (CI)***	***FC***	***P value***
**CLL cell count [x10^9/L]**	2.21 (0.96–3.46)	1.37 (0.85–1.89)	-0.32	0.389
**Classical monocytes (MON)**				
% of MON	77.4 (71.8–83.0)	82.2 (79.1–85.3)	0.07	**0.038**
Absolute count [x10^9/L]	0.27 (0.19–0.35)	0.33 (0.28–0.39)	0.23	0.212
HLA-DR MFI	274 (211–337)	357 (302–411)	0.32	0.099
CD64 MFI	338 (287–390)	352 (315–389)	-0.06	0.914
CD11b MFI	689 (557–820)	744 (656–833)	0.11	0.503
**Intermediate MON**				
% of MON	10.4 (7.94–12.9)	8.84 (7.37–10.3)	-0.34	0.207
Cell count [x10^9/L]	0.03 (0.02–0.04)	0.04 (0.03–0.04)	0.23	0.666
HLA-DR MFI	1517 (1042–1992)	1998 (1756–2240)	0.50	**0.012**
CD64 MFI	313 (253–373)	329 (296–632)	0.12	0.379
CD11b MFI	580 (468–692)	597 (536–658)	0.11	0.532
**Non-classical MON**				
% of MON	12.2 (8.25–16.1)	8.03 (6.36–9.70)	-0.23	**0.046**
Cell count [x10^9/L]	0.03 (0.02–0.04)	0.03 (0.02–0.03)	-0.17	0.419
HLA-DR MFI	778 (510–1045)	1011 (823–1198)	0.53	**0.028**
CD64 MFI	162 (144–180)	157 (147–167)	-0.04	0.416
CD11b MFI	108 (77.3–139)	116 (103–129)	0.33	0.093

**Neutrophils**				
Cell count [x10^9/L]	3.02 (2.24–3.79)	3.05 (2.59–3.51)	0.01	0.907
CD64 MFI	89.1 (77.0–101)	95.6 (84.3–107)	0.02	0.439
CD54 MFI	18.5 (15.9–21.2)	21.0 (18.3–23.8)	0.10	0.225
CD11b MFI	145 (96.1–194)	179 (144–214)	0.12	0.247
CD62L MFI	452 (278–626)	548 (449–648)	0.01	0.288
**NK cells**				
Cell count [x10^9/L]	0.17 (0.12–0.21)	0.17 (0.12–0.22)	0.03	0.577
HLA-DR MFI	62.4 (34.2–90.5)	10.8 (31.6–110)	-0.27	0.267
CD69 MFI	46.8 (30.9–62.0)	35.3 (30.3–40.3)	-0.25	0.538
**T cells**				
Cell count [x10^9/L]	1.34 (0.95–1.76)	1.64 (1.27–2.00)	0.21	0.496
CD4+/CD8+ ratio	1.32 (0.79–1.85)	1.05 (0.80–1.30)	-0.23	0.317
HLA-DR (MFI) on CD4+ cells	29.6 (24.5–34.6)	31.4 (26.5–36.3)	-0.06	0.701
HLA-DR (MFI) on CD8+ cells	45.2 (36.9–53.4)	48.1 (41.9–54.3)	0.13	0.629
% **Treg cells** of CD4+ cells	12.0 (9.23–14.8)	6.70 (5.50–7.89)	-0.45	**< 0.001**
Cell count [x10^9/L]	0.07 (0.04–0.11)	0.04 (0.03–0.05)	-0.43	0.062

CI, confidence interval; MFI, mean fluorescence intensity.

Table S7. Comparison of immune subset percentages and activation markers expressed on immune cells in CLL patients treated with ibrutinib, idelalisib and venetoclax.

**Populations/markers**	**Ibrutinib (IBR)** ***Mean (CI)***	**Idelalisib (IDEL)** ***Mean (CI)***	**Venetoclax (VEN)** ***Mean (CI)***	***P value IBR vs IDEL***	***P value IBR vs VEN***	***P value IDEL vs VEN***
**CLL cells**						
Cell count [x10^9/L]	4.98 (2.23–7.73)	2.52 (0.46–4.58)	0.02 (0.01–0.03)	0.324	**<0.001**	**<0.001**
HLA-DR MFI	651 (513–789)	600 (386–814)	1190 (649–1731)	0.567	0.178	0.209
% CD27+	75.5 (69.1–81.9)	70.6 (58.0–83.1)	60.3 (17.5–99.5)	0.639	0.710	0.916
% CD38+	47.2 (35.4–59.0)	30.8 (23.1–38.4)	69.1 (35.9–102)	0.345	0.181	0.161
% CD49d+	58.2 (43.7–72.8)	53.5 (11.1–95.9)	97.8 (76.2–119)	0.706	0.133	0.286
**Classical monocytes (MON)**						
% of MON	83.1 (79.1–87.0)	80.5 (76.6–84.4)	87.9 (83.2–92.6)	0.065	0.581	**0.048**
Cell count [x10^9/L]	0.35 (0.27–0.44)	0.29 (0.20–0.38)	0.32 (0.15–0.49)	0.402	0.705	0.806
HLA-DR MFI	339 (270–408)	274 (289–459)	348 (242–455)	0.287	0.398	0.806
CD64 MFI	339 (300–379)	408 (331–486)	284 (227–341)	0.118	0.441	0.093
CD11b MFI	753 (674–832)	762 (551–974)	551 (314–787)	0.936	0.192	0.216
**Intermediate MON**						
% of MON	7.79 (6.35–9.23)	11.3 (8.71–13.9)	4.81 (2.74–6.88)	**0.002**	0.103	**0.006**
Cell count [x10^9/L]	0.03 (0.02–0.04)	0.04 (0.02–0.07)	0.02 (0.01–0.04)	0.568	0.483	0.108
HLA-DR MFI	1676 (1372–1980)	2163 (1788–2537)	2090 (1411–2770)	**0.007**	**0.049**	0.971
CD64 MFI	308 (269–348)	368 (306–430)	271 (210–332)	**0.018**	0.496	**0.046**
CD11b MFI	600 (533–667)	590 (471–709)	456 (371–540)	0.857	0.070	0.221
**Non-classical MON**						
% of MON	7.10 (5.43–8.77)	8.24 (5.71–10.8)	7.26 (3.45–11.1)	0.375	0.522	0.704
Cell count [x10^9/L]	0.03 (0.02–0.03)	0.03 (0.02–0.04)	0.03 (0.01–0.05)	0.878	0.996	0.972
HLA-DR MFI	949 (698–1201)	931 (803–1059)	719 (457–980)	0.364	0.274	0.158
CD64 MFI	153 (141–165)	168 (149–187)	144 (129–158)	0.087	0.998	0.259
CD11b MFI	125 (109–141)	99.7 (86.1–113)	83.5 (43.8–123)	0.075	**0.049**	0.200
**Neutrophils**						
Cell count [x10^9/L]	3.19 (2.65–3.73)	2.50 (1.58–3.43)	2.31 (0.90–3.71)	0.069	0.213	0.649
CD64 MFI	96.0 (82.6–109)	95.7 (73.7–118)	84.1 (74.3–93.9)	0.871	0.280	0.723

CD54 MFI	21.5 (17.2–25.9)	21.3 (18.2–24.3)	25.7 (16.7–34.7)	0.258	0.358	0.990
CD11b MFI	163 (132–193)	179 (94.7–263)	221 (145–297)	0.910	0.195	0.727
CD62L MFI	519 (390–648)	547 (372–721)	341 (201–482)	0.377	0.192	0.085
**NK cells**						
Cell count [x10^9/L]	0.14 (0.11–0.18)	0.20 (0.11–0.29)	0.18 (0.10–0.28)	0.444	0.732	0.993
HLA-DR MFI	47.2 (27.6–66.8)	82.2 (0.79–165)	49.0 (20.0–78.0)	0.685	0.552	0.219
CD69 MFI	38.0 (28.7–47.3)	45.4 (16.8–74.0)	n.a.	0.904	n.a.	n.a.
**T cells**						
Cell count [x10^9/L]	1.61 (1.13–2.09)	1.97 (1.12–2.82)	1.10 (0.61–1.57)	0.299	0.139	0.095
CD4+/CD8+ ratio	0.92 (0.75–1.09)	0.96 (0.37–1.55)	1.49 (0.47–2.51)	0.255	0.170	0.166
HLA-DR (MFI) on CD4+ cells	30.7 (25.7–35.7)	30.8 (21.0–40.6)	31.8 (17.0–46.4)	0.618	0.516	0.441
HLA-DR (MFI) on CD8+ cells	45.8 (37.8–53.9)	47.7 (37.9–57.5)	46.8 (32.2–61.3)	0.488	0.225	0.824
% **Treg cells** of CD4+ cells	7.50 (6.07–8.93)	6.80 (4.12–9.48)	4.31 (2.18–6.44)	0.256	0.070	0.173
Cell count [x10^9/L]	0.05 (0.03–0.06)	0.03 (0.02–0.05)	0.04 (0.02–0.06)	0.146	0.828	0.896

n.a., not available (CD69 was not measured in venetoclax-treated patients); IBR, ibrutinib; IDEL, idelalisib; VEN, venetoclax; CI, confidence interval; MFI, mean fluorescence intensity.

Patient similarity network (PSN) analysis

The use of networks to analyse multivariate data is based on constructing a network from vector data. This construction uses a similarity between each pair of vectors in the dataset. In a patient network case, each vector describes one patient represented by one network vertex; components of that vector represent markers used.

The constructed network can be visualised using one of the algorithms, which allows displaying the pair-to-pair relationships between patients in a 2D layout (for example, onscreen). In this layout, such vertices that are sufficiently similar are connected by ties. In our case, one Patient Similarity Network (PSN) vertex represents one patient (later, the term *patient* is used instead of the term *vertex*). A natural consequence of the similarity between patients in the network is that similar patients are close to each other in the network layout. Conversely, dissimilar patients are in distant parts of the network layout.

Because of the similarities, groups (clusters) of similar patients can be identified in the network. Such clusters can be identified not only visually but also automatically. To detect non-overlapping clusters, we use the Louvain method [S1], which is based on the optimisation of the so-called modularity. Modularity measures the strength of the network division into the clusters. The advantage of using methods to detect clusters in networks is that there is no need to estimate their number in advance, and the result can be clusters that differ significantly in size.

If patients in a cluster are highly similar, then the cluster is dense and is visually separated from the rest of the network. The more densely interconnected and more separated the cluster is from its surroundings, the more specific and unambiguously it can be interpreted as a common profile of patients in this cluster. Such a profile can be obtained as a vector representing a virtual (average) patient having individual markers equal to the arithmetic averages of the markers for all patients in the cluster.

The quality of such a profile can then be assessed from two perspectives. The first aspect is the confidence intervals of the averages of markers of all patients in the cluster. The second aspect is the degree of unambiguity with which patients are included in individual clusters. For this purpose, the so-called silhouette is used in data mining [S2], the value of which is from -1 to 1. The closer the value of the silhouette for the individual patient is to 1, the more clearly the patient is a member of the cluster to which they belong. However, in real datasets, there are situations in which some patients may not be clearly assigned. In this case, their silhouettes may be less than 0. This does not mean that they are incorrectly assigned to the cluster; it only shows that such patients could also be assigned to another cluster. Therefore, generally, if the silhouette values are positive for all patients in the cluster (the higher the values, the better), then the cluster defines a clear common profile of the patients in the cluster. Conversely, the more negative values of the silhouette in the cluster, the more problematic is the perception of this profile as an unambiguous characteristic.

The basis for the above considerations is the PSN construction from vector patient data. Networks can be constructed not only by different algorithms with different settings but also from different subsets of studied markers. Generally, the key is to find a network that is good enough both in terms of modularity (separable clusters) and in terms of silhouette (unambiguous assignment of the patients). Therefore, our methodology is based on the automatic generation of different networks, automatically balancing the relationship between modularity and silhouette. All networks used in our manuscript were constructed by the LRNet algorithm [S3] and selected using the application of this methodology.

Use of network layout to analyse trends

Normally, clusters in networks are distributed in more complex structures, where each cluster is in the layout adjacent to several other clusters. In both networks constructed from the vector

data in our study, it can be seen that the clusters are arranged almost linearly, which means that they gradually follow each other. For example, if we display the layout horizontally, the clusters follow each other from left to right. Figure 3A (in the main manuscript) shows the network layout with coloured clusters. It can be seen from the silhouette in the column chart in Figure S1, patients in the first (blue) and third (green) clusters are least unambiguously included. This is because these two clusters cannot be easily separated from the others (to a lesser extent, this also applies to the other two clusters). However, the profiles of individual clusters (see the right side of Figure 3A in the main manuscript) and their visualisation in the layout show differences in the immune cell activation, CLL cell counts, as well as treatment strategy. This, in most cases, corresponds well to the clusters detected.

Using visualisation, we can add one extra marker to each patient, related to its horizontal position in the network layout. In our case, it is an x-coordinate, where we assigned the x-coordinate equal to zero to the centre of the layout; patients to the left of this centre have a negative x-coordinate, and patients to the right have a positive x-coordinate. The unit on the x-axis, in our case, is a pixel. Figure 4A (in the main manuscript) shows scatterplots expressing the relationships between selected markers and the patient's horizontal position in the layout. In each scatterplot, and in the network layout in Figure 4B (in the main manuscript), a linear trend emphasises the relationship between the patient's position in the PSN layout and the activation rate and treatment, respectively.

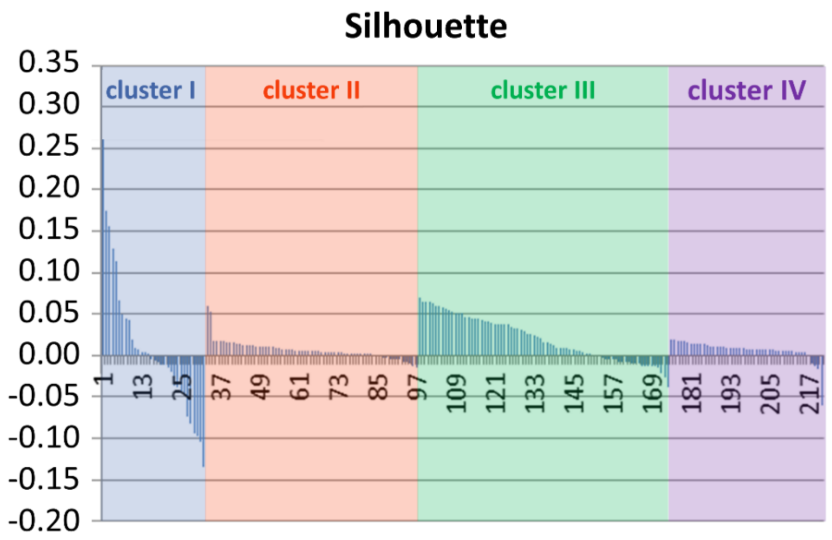

Figure S1. The silhouette of clusters detected in PSN (presented in Figure 3A in the main manuscript) based on immune cell activation markers in CLL patients. The silhouette shows that most patients were correctly assigned to the individual clusters. The y-axis represents the silhouette value; the x-axis represents patients in individual clusters ordered by decreasing silhouette value.

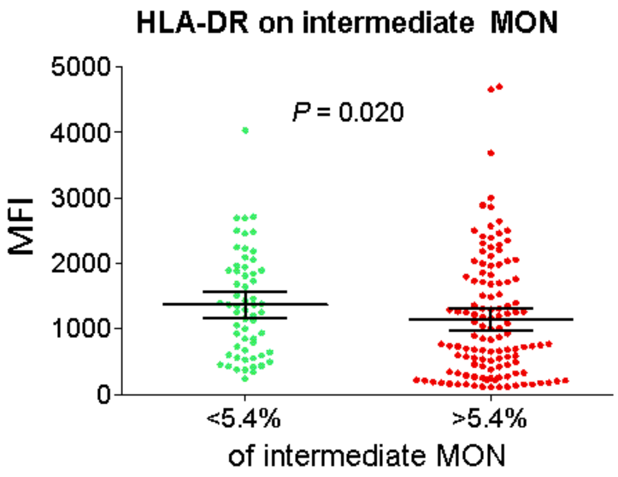

Figure S2. The difference in HLA-DR expression on intermediate monocytes (MON) between CLL patients with lower and higher numbers of intermediate monocytes (cut-off 5.4%).

References

S1. Blondel, V. D., Guillaume, J. L., Lambiotte, R. & Lefebvre, E. Fast unfolding of communities in large networks. *J. Stat. Mech.* **10**, P10008 (2008).

S2. Rousseeuw, P. J. Silhouettes: a graphical aid to the interpretation and validation of cluster analysis. *J. Comput. Appl. Math.* **20**, 53–65 (1987).

S3. Ochodkova, E., Zehnalova, S. & Kudelka, M. Graph construction based on local representativeness. *Lecture Notes in Computer Science.* **10392**, 654–665 (2017).
